# Erythroderma Triggered by Cutaneous Fungal Infection and Prolonged Steroid Use: A Lesson Learned

**DOI:** 10.1002/kjm2.70012

**Published:** 2025-03-24

**Authors:** Chin‐Yu Lee, Wei‐Cheng Fang, Yue‐Chiu Su, Yang‐Yi Chen

**Affiliations:** ^1^ Department of Dermatology Kaohsiung Medical University Hospital, Kaohsiung Medical University Kaohsiung Taiwan; ^2^ College of Medicine Kaohsiung Medical University Kaohsiung Taiwan; ^3^ Department of Dermatology Kaohsiung Municipal Siaogang Hospital Kaohsiung Taiwan; ^4^ Department of Pathology Kaohsiung Medical University Kaohsiung Taiwan; ^5^ Graduate Institute of Clinical Medicine, College of Medicine Kaohsiung Medical University Kaohsiung Taiwan

1

A 68‐year‐old male with idiopathic pulmonary fibrosis (IPF) initially presented with generalized folliculitis and received oral doxycycline for 2 months, showing only partial improvement (Figure 1a–c). Concurrently, he began Treprostinil inhalation therapy for IPF but experienced an allergic reaction with anterior neck swelling, necessitating oral prednisolone (10–15 mg/day for 4 weeks). His skin condition rapidly progressed to diffuse erythematous pruritic papulonodules with excoriation and scaling across the trunk, back, face, neck, and extremities without significant nail changes (Figure 1d–f). Despite continued systemic steroid treatment (prednisolone 10 mg/day for 2 weeks, followed by intravenous methylprednisolone 80 mg/day for 3 days), the condition worsened, resulting in erythroderma (Figure 1g–i).

**FIGURE 1:**
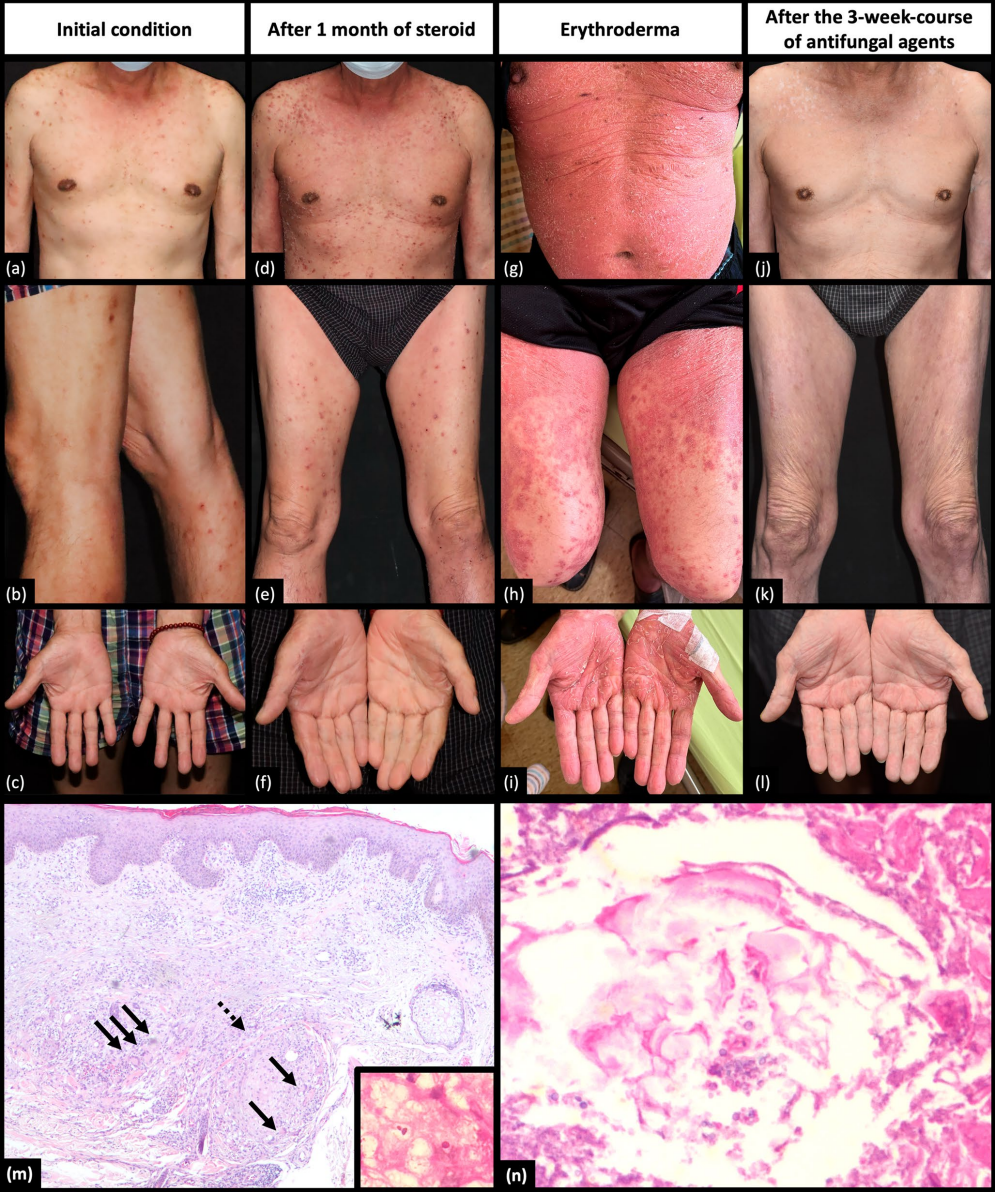
Clinical images of the patient (a–c) before the episode, (d–f) after steroid use for 1 month, (g–i) erythroderma, and (j–l) after the 3‐week‐course of terbinafine, (m) Dilated hair follicle with perifollicular granulomatous infiltration (dotted arrow) and multinucleated giant cell (fine arrow) in the dermis, H&E stain, ×40, Inset: Fungal spores around the granulomatous infiltrates noted in the PAS stain, (n) Fungal spores in the hair follicle, PAS stain, ×200.

A skin biopsy revealed fungal spores within hair follicles, perifollicular granulomatous infiltration with multinucleated giant cells in the deep dermis, and features of eczematous dermatitis. Periodic acid‐Schiff staining confirmed numerous spores within the hair follicles and the dermis around the granulomatous infiltrates (Figure 1m,n). Laboratory tests showed elevated C‐reactive protein levels and significant eosinophilia (eosinophil count: 19,881/mm^3^).

Given the evidence of a cutaneous fungal infection, oral itraconazole (400 mg/day) was added to systemic methylprednisolone (80 mg/day). The erythroderma showed marked improvement, with a resolution of eosinophilia (eosinophil count: 155.3/mm^3^) after 5 days of combined therapy. Due to persistent hiccups and nausea, itraconazole was discontinued after 5 days and substituted with terbinafine (250 mg/day), and the steroid was tapered. Total clearance was observed after 3 weeks of treatment without recurrence of skin lesions (Figure 1j–l). The clinical presentation, pathological findings, and treatment response confirmed a diagnosis of cutaneous fungal infection‐induced erythroderma.

Erythroderma, defined as extensive erythema affecting over 90% of the body surface, has multiple etiologies, such as inflammatory skin disorders, malignancies, and infections [1]. Identifying the cause of erythroderma can be challenging. Therefore, the patient's personal and medical history, physical examination, skin biopsy, laboratory survey, and microbiome cultures may provide further information and are crucial for surveying the cause of erythroderma [2].

Cutaneous fungal infections represent a relatively uncommon cause of erythroderma, which is predominantly associated with dermatophyte infections (Table S1) [1, 2, 3, 4, 5]. Several of the reported cases have a prior history of the use of systemic or topical corticosteroids before developing erythroderma. In our case, given the lack of other signs of fungal infection, a fungal culture was not performed, resulting in a delayed diagnosis; prolonged systemic corticosteroids weakened the host defense against the microbiome, thereby enabling fungal proliferation and extensive skin involvement [3]. It highlights the importance of routine fungal testing in refractory or worsening skin conditions, especially in patients on systemic corticosteroids. For erythrodermic patients with a prolonged history of steroid use, previous clinical presentations of refractory folliculitis, hyperkeratotic or yellowish nails, or poor response to steroid treatment for erythroderma, clinicians should consider fungal infections as a primary differential diagnosis.

This case emphasizes the importance of considering fungal etiologies in erythroderma. It illustrates the hazards of excessive systemic corticosteroid use in promoting fungal proliferation and worsening status. Early diagnosis and prompt antifungal therapy are essential for effective management and improving outcomes.

## Conflicts of Interest

The authors declare no conflicts of interest.

## Supporting information


**Supplementary Table 1:** Reported erythroderma cases due to cutaneous fungal infection.

## Data Availability

Data sharing not applicable to this article as no datasets were generated or analysed during the current study.
