# Supplementary material for: Erythroderma Triggered by Cutaneous Fungal Infection and Prolonged Steroid Use: A Lesson Learned
Source: Kaohsiung J Med Sci. 2025 Mar 24;41(6):e70012. doi: 10.1002/kjm2.70012 (PMC12199569; doi:10.1002/kjm2.70012)
Supplement: Supplementary file 1 — Supplementary Table 1: Reported erythroderma cases due to cutaneous fungal infection. [file KJM2-41-e70012-s001.docx]

**Supplementary Table 1** **Reported erythroderma cases due to cutaneous fungal infection**

| Author | Sex | Age | Underlying disease | Disease duration | Fungal culture result | Previous treatment | Systemic treatment | Topical treatment | Time to resolution |
| --- | --- | --- | --- | --- | --- | --- | --- | --- | --- |
| Das (2022)^1^ | M | 30 | None | 4 years | *T. mentagrophytes* | IM triamcinolone | Oral itraconazole 100mg twice daily | luliconazole cream | 4 months |
|  | M | 41 | None | 5 years | *T. rubrum* | Clobetasol cream, betamethasone cream, antifungal soaps | Oral itraconazole 100mg twice daily | luliconazole cream | 4 months |
|  | M | 21 | None | 7 months | Not performed | Oral fluconazole, clobetasol cream, halobetasol cream | Oral itraconazole 100mg twice daily | luliconazole cream | 3 months |
|  | M | 46 | HTN, DM | 2 years | Not performed | IM triamcinolone, oral prednisolone, oral itraconazole, betamethasone cream | Oral terbinafine 250 mg twice daily | luliconazole cream | 7 months |
|  | M | 38 | DM | 6 months | *T. mentagrophytes* | Oral fluconazole, halobetasol cream | Oral itraconazole 100mg twice daily | luliconazole cream | 6 months |
|  | M | 58 | HTN, CKD | 3 years | *T. mentagrophytes* | Betamethasone cream, clobetasol cream, antifungal soaps | Oral fluconazole 150 mg alternate days | amorolfine cream | 3 months |
|  | F | 33 | None | 1 year | *T. mentagrophytes* | Clobetasol cream, betamethasone cream, antifungal soaps | Oral itraconazole 100mg twice daily | sertaconazole cream | 3 months |
|  | M | 48 | HIV, HTN | 8 months | *T. mentagrophytes* | Oral fluconazole, halobetasol cream | Oral fluconazole 150 mg twice weekly | luliconazole cream | 4 months |
|  | M | 28 | DM | 1 year | *T. mentagrophytes* | Oral fluconazole, oral terbinafine, halobetasol cream, clobetasol cream | Oral itraconazole 100mg twice daily | luliconazole cream | 4 months |
|  | F | 41 | None | 5 years | *T. mentagrophytes* | Oral terbinafine, clobetasol ream, antifungal soaps | Oral itraconazole 100mg twice daily | sertaconazole cream | 4 months |
|  | F | 35 | None | 6 months | Not performed | Oral terbinafine, clobetasol ream, antifungal soaps | Oral itraconazole 100mg twice daily | sertaconazole cream | 3 months |
|  | M | 72 | HTN, DM | 2 years | *T. mentagrophytes* | IM triamcinolone, oral methylprednisolone, oral terbinafine, clobetasol cream | Oral terbinafine 250 mg twice daily | sertaconazole cream | 8 months |
|  | F | 44 | None | 6 months | *T. mentagrophytes* | Oral prednisolone, oral fluconazole, halobetasol cream, antifungal soaps | Oral itraconazole 100 mg twice daily | luliconazole cream | 6 months |
|  | M | 48 | CVA | 9 months | *T. rubrum* | IM triamcinolone; clobetasol cream | Oral itraconazole 100 mg twice daily | benzoic/salicylic acid ointment, luliconazole cream, ketoconazole  shampoo | 5 months |
|  | M | 52 | HIV, osteoarthritis | 4 months | *E. floccosum* | Probable oral corticosteroid, antiscabetic treatment | Oral itraconazole twice daily | benzoic/salicylic acid ointment, luliconazole cream | 5 months |
|  | M | 22 | None | 1.5 years | *T. mentagrophytes* | Clobetasol cream | Oral itraconazole 100mg twice daily | Ciclopirox shampoo | 3 months |
|  | F | 31 | RA, DM | 1 year | *T. mentagrophytes* | Oral prednisolone | Oral itraconazole twice daily | benzoic/salicylic acid ointment, luliconazole cream | 6 months |
| El-Darouti (2013)^2^ | M | 16 | Not mentioned | Not mentioned | *T. violaceum* | None | Griseofulvin and itraconazole 150mg twice daily | - | 1 month  (marked improvement within 5 days and virtual clearing of the erythroderma after 1 month), no recurrence |
| Gupta (2000)^3^ | F | 66 | Not mentioned | 1 month | Not performed | triamcinolone acetonide 0.1% ointment topically with cyproheptadine  hydrochloride 2 mg orally daily | Fluconazole 150 mg orally daily, cefuroxime  250 mg twice daily | Miconazole 2% cream | 20 days, no recurrence |
| Hidayah (2021)^4^ | M | 39 | None | 10 years | *T. rubrum* | Oral corticosteroid for 10 years, topical antifungal | Two pulse of 1-week of 200 mg itraconazole twice a day for each month | 2% ketoconazole, 10% urea lotion | 29 days  (clinical improvement within 7 days, significant improvement after 29 days) |
| Kumar (2024)^5^ | M | 55 | Not mentioned | 1 year | Not performed | topical/oral steroids either alone or in combination with topical antifungal agents | Oral itraconazole 100 mg twice a day | clotrimazole 1 w/w% cream | 12 weeks |
| Lyra (2017)^6^ | M | 45 | Chronic alcoholism | 2 years | *T. tonsurans* | Not mentioned | Oral itraconazole 200 mg daily | - | 28 days |
| Si (2023)^7^ | M | 48 | Myasthenia gravis | 1 month | *T. rubrum* | systemic prednisolone daily for 10 years, occasional topical antifungals, tacrolimus for 2 months, intermittent topical hormones | Initially 200 mg oral itraconazole twice daily, shifted to 0.2 g intravenous voriconazole twice daily, oral terbinafine 250 mg once daily | bifonazole cream | 1 month  (partially subsided within 14 days, significant diminished after 1 month) |
| Yousefian (2020)^8^ | M | 39 | None | 6 years | Not performed | betamethasone 0.05% cream and triamcinolone 0.1% ointment | Oral fluconazole 75 mg per week for six weeks | Ketoconazole 2% shampoo and cream | 6 weeks |
| Our case | M | 68 | Idiopathic pulmonary fibrosis | 2 months | Not performed | Oral corticosteroid, oral doxycycline, topical corticosteroid, topical clindamycin | Oral itraconazole 400mg daily, intravenous methylprednisolone 80mg daily | Betamethasone ointment, urea cream | 3 weeks  (marked improvement within 5 days and total clearing of the erythroderma after 3 weeks) |

T. mentagrophytes, Trichophyton mentagrophytes; IM, intramuscular; T. rubrum, Trichophyton rubrum; HTN, hypertension; DM, diabetes mellitus; CKD, chronic kidney disease; CVA, cerebrovascular accident; HIV, human immunodeficiency virus infection; E. floccosum, Epidermophyton floccosum; RA, rheumatoid arthritis; T. violaceum, Trichophyton violaceum; T. tonsurans, Trichophyton tonsurans

**References**

1. Das A, Sil A, Jaiswal S, Agarwal A, Das K. Erythrodermic dermatophytosis: an alarming consequence of steroid abus e and misuse. A multicentre prospective study from India. *Clinical and experimental dermatology* 2022;47:1735-8.

2. El-Darouti MA. An Extremely Rare Cause of Erythroderma. In: El-Darouti MA, ed. *Challenging Cases in Dermatology*. London: Springer London, 2013:371-8.

3. Gupta R, Khera V. Erythroderma due to dermatophyte. *Acta dermato-venereologica* 2001;81:70.

4. Hidayah RMN, Anjani AD, Ramali LM, Suwarsa O, Gunawan H. Exfoliative dermatitis due to dermatophytosis. *Journal of infection in developing countries* 2021;15:306-9.

5. Kumar L, Mittal A, Balai M, Solanki NK, Sehgal S. Inappropriate Use of Steroids in Superficial Dermatophytosis: An Uncom mon Case of Erythroderma. *The Journal of the Association of Physicians of India* 2024;72:107.

6. Lyra MR, Muniz Álvarez B, Lanziano AL, et al. Exfoliative erythroderma and palmoplantar hyperkeratosis associated wi th Majocchi's granuloma by Trichophyton tonsurans in a patient with AI DS. *Revista iberoamericana de micologia* 2017;34:185-8.

7. Si H, Li Y, Huang Z, Cui Y, Li S. Erythroderma combined with deeper dermal dermatophytosis due to Tricho phyton rubrum in a patient with myasthenia gravis: first case report a nd literature review. *BMC infectious diseases* 2023;23:789.

8. Yousefian F, Crowley C, Skupsky H, Calame A, Cohen PR. Tinea Corporis-associated Erythroderma: Case Report and Review of Eryt hrodermic Patients with Chronic Dermatophyte Infection. *Cureus* 2020;12:e7578.
